# Prevalence and correlates of suicidal behavior in primary care settings in Mozambique

**DOI:** 10.1186/s12888-022-04059-y

**Published:** 2022-06-23

**Authors:** Vasco F. J. Cumbe, Maria Nélia Manaca, Dana L. Atkins, Alberto Muanido, Leecreesha Hicks, Maria A. Oquendo, Jair de Jesus Mari, Bradley H. Wagenaar

**Affiliations:** 1 Mental Health Department, Ministry of Health, Provincial Health Directorate of Sofala, Beira, Mozambique; 2 Mental Health and Psychiatry Department, Faculty of Medicine, Eduardo Mondlane University (UEM), Maputo, Mozambique; 3 Medicine Department, Psychiatry and Mental Health Service, Beira Central Hospital, Sofala, Beira, Mozambique; 4 Departamento de Psiquiatria, Escola Paulista de Medicina, Universidade Federal de São Paulo, UNIFESP, São Paulo, Brazil; 5 Health Alliance International, Beira, Mozambique; 6 Department of Global Health, University of Washington, Seattle, WA USA; 7 Health Alliance International, Seattle, WA USA; 8 Department of Psychiatry, Perelman School of Medicine, University of Pennsylvania, Philadelphia, PA USA; 9 Department of Epidemiology, University of Washington, Seattle, WA USA

**Keywords:** Suicidal behavior, Suicidal ideation, Suicidal plan, Suicide attempt, Primary health care, Mozambique

## Abstract

**Background:**

This study assessed the prevalence of suicidal behavior and associated risk factors in public primary health care in Mozambique.

**Methods:**

The Mini International Neuropsychiatric Interview was used to evaluate suicidal behavior among 502 adults attending three Primary Health Care (PHC) settings.

**Results:**

In the past month, 13% (*n* = 63) of PHC attendees expressed suicidal ideation, 8% (*n* = 40) had made a suicide plan, 4% (*n* = 20) had made a suicide attempt, and 5% (*n* = 25) reported a lifetime suicide attempt. Females had 2.8-fold increased odds of suicide plan (95% CI: 1.5, 5.5) and 3.3-fold increased odds of suicide attempt in the past month (95% CI: 1.2, 9.1). Each 10-year increase in age was associated with 0.61-fold the odds of suicide plan (95% CI: 0.38, 0.98) and 0.09-fold the odds of suicide attempt (95% CI: 0.01, 0.69) in the past month. People living with HIV (PLWHA) had 2.2-fold increased adjusted odds of past month suicide attempt (CI: 1.1, 4.1).

**Conclusion:**

Suicidal behaviors are common among adults attending PHC clinics in Mozambique. Screening and linkage to effective preventive interventions are urgently needed in PHC settings. Females, younger individuals, and PLWHA are at elevated risk for suicidal behavior in PHC.

## Introduction

Suicide is a serious global public health issue and is among the top twenty leading causes of death worldwide, with more deaths due to suicide than malaria, breast cancer, or war and homicide [1–3]. The reduction of suicide mortality is an important priority of the World Health Organization (WHO) and is included as an indicator in the United Nations Sustainable Development Goals (SDGs) [4] and in the WHO Mental Health Action Plan 2013–2030 [5]. About 800,000 people die from suicide annually [6–8], representing a global mortality rate of 16 people per 100,000 population or one death every 40 s [6]. Due to the COVID-19 pandemic and associated mental health concerns, there is global concern that suicidal behavior may increase during and after pandemic [9]. Worldwide, suicide is the second leading cause of premature mortality in people aged 15—29 years after traffic accidents and the third leading cause for those between 15—44 years old [8, 10].

Due to stigma around suicidal behavior, estimates of suicidal behavior may be largely underestimated in some countries – especially in countries where suicide is illegal [11]. The WHO estimates that for each suicide, there are more than 20 suicide attempts. Suicide occurs globally, although 79% of all global suicides occur in low- and middle-income countries (LMICs) [12]. Rates of suicidal ideation, suicide planning and suicide attempts in the last year show significant variability across LMICs in patients attending in primary health care. One study carried out in five LMICs (India, Nepal, Ethiopia, Uganda, South Africa) found that an average of 10.3% of those who sought primary health care reported suicidal ideation in the past 12 months [13]. This study found large variability, however, with suicidal ideation ranging from 14.8% of primary care attendees in South Africa to 5.0% in Uganda. During the last 12 months, in Nepal and South Africa, younger individuals had increased prevalence of suicidal ideation and in Ethiopia and Nepal, females had double the prevalence of suicidal ideation in primary health care outpatient settings [13]. Another study on suicidal ideation in Morocco reported a rate of 5.3% per year [14]. A complicating factor in evaluating and comparing these statistics across countries is a lack of uniformity in the assessment and reporting of suicidal thoughts and behaviors. For example, a study conducted in one Kenyan general medicine outpatient facility assessed frequency of suicidal ideation and suicide attempts in the previous month instead of the previous 12. It found that the rate of suicidal ideation was 20%, suicide planning was 10%, and that 4% of participants reported a suicide attempt [15]. This creates some challenges when comparing to a study in Morocco that found 1.2% of those surveyed had attempted suicide in the previous 12 months [14].

In Mozambique, over 90% of the population receives health care through the centralized public-sector Ministry of Health system of over more 1,300 public clinics [16]. The centralized nature of the Mozambican health system presents an opportunity to integrate screening for suicidal behavioral in routine activities as a strategy for suicide prevention. In 2014, the WHO estimated Mozambique to have an age-standardized suicide rate of 27.4/100,000, which put Mozambique’s suicide rate at a significantly higher rate than the global average and made it the highest rate in Africa [8]. However, in 2019, the WHO revised this estimate to 8.4/100,000 citing concerns about “data quality” of suicide estimates for Mozambique and noted that these numbers may not be reliable. It would not be surprising if the prevalence of suicidal behavior in Mozambique were greatly elevated considering that its population has endured decades of trauma due to anti-colonial struggles (1964—1975) and another protracted war (1976 to 1992), which lead to equally protracted political instability and destruction of health systems. These historical traumas have been compounded by severe natural disasters such as cyclones Idai and Kenneth in 2019 which left hundreds of thousands of people homeless and contributed to widespread destruction of the health system [17].

Mozambique has generated limited research on suicidal behavior; a literature search uncovered only three peer-reviewed publications focused on suicide in the country. The first study conducted in legal medicine settings on deaths due to suicide in Sofala Province showed that 10% of over 1,000 violent deaths were due to suicide, with a mean age of 31 years. The most common methods were hanging (43%) followed by use of unspecified substance (28%) and use of rat poison (26%) [18]. In the same study, data on suicide attempts showed that 18% of emergency room psychiatric visits were for suicide attempts, that females represented the majority (68%), and the mean age was 27 years. The most common method for attempts was rat poison (66% of attempts) [18]. Another population-based household survey done in central Mozambique (3,060 individuals across Manica and Sofala Provinces) reported a 30-day prevalence of suicidal ideation of 5.9% [19]. The third study reported on patients with suicide attempts treated at Nampula Central Hospital from 2014 to 2016, in which more than half of the patients were aged 15—24 years old and suggested that impulsivity was the main pathway to suicidal behavior [20].

To date, there are no previous studies of suicidal behavior amongst primary care populations in Mozambique. Primary health care in this context provides a range of care, including co-located but specialized mental health services. Suicidal behavior and other mental health problems are not routinely tracked among primary healthcare attendees but instead individuals seen as needing mental health services are referred to co-located mental health specialists. The lack of integration of mental health within primary healthcare – including routine screening for suicidal behavior – constitutes a great missed opportunity for suicide prevention and treatment in Mozambique. Primary care settings are often seen as an optimal setting to screen at-risk patients for suicidal ideation and link patients to suicide prevention interventions. Identifying these high-risk individuals and providing them with follow-up care and support should be a key component of all comprehensive suicide prevention strategies evaluation of suicide prevention strategies. The aim of this study is to provide information about the prevalence of suicidal behavior and associated factors for adults attending primary care clinics in Mozambique. Information from this study can aid in the development and implementation of screening, identification, and suicide prevention interventions.

## Methods

### Study setting and participants

**Fig. 1:**
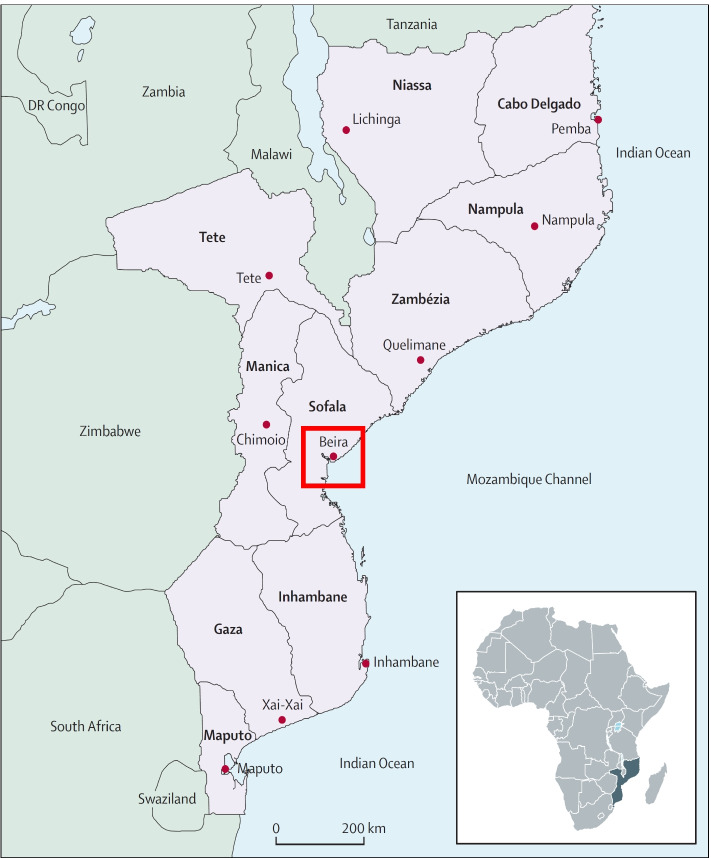
Political map of Mozambique including provincial capital cities. Focal area of Beira City and surrounding Dondo highlighted by the red box. Figure sourced from: Fernandes QF, Wagenaar BH, Anselmi L, Pfeiffer J, Gloyd S, Sherr K. Effects of health-system strengthening on under-5, infant, and neonatal mortality: 11-year provincial-level time-series analyses in Mozambique [23]

This study was conducted in Sofala Province (see Fig. 1), located in the central region of Mozambique, with a population of approximately 2.4 million. The official language is Portuguese, but the most common languages spoken in rural areas are ​​Cisena and Cindau. Sofala has a literacy rate of 56%, an infant mortality rate of 62 per 1,000 live births, a life expectancy of 57 years, and an HIV prevalence of 14% [21]. Sofala province has 166 health facilities, of which 27 (16%) have trained mental health staff. These staff include 3 Psychiatrists, 37 Clinical Psychologists, 42 Psychiatric Technicians (mid-level health professionals specialized in mental health care), 1 Social Worker, and 3 Occupational Therapist [22]. The present study was conducted across three health facilities: two in Beira City (Macurungo and Chingussura), and one in Dondo (Dondo Health Facility). Beira is the capital of Sofala Province and the second largest city in Mozambique after the national capital of Maputo. Beira City has a population of approximately 600,000 individuals with a health system comprised of 13 health facilities and 1 quaternary level Central Hospital. Dondo is the closest city to Beira (35 km), with 8 primary care health facilities serving a population of approximately 210,000 [21]. We selected the above-mentioned three facilities for this study, representative of a typical primary health care in Mozambique, because they: 1) had at least 1 psychiatric technician and 1 clinical psychologist; 2) were high-volume facilities providing general primary healthcare; 3) provided comprehensive maternal and child healthcare (to capture women in the perinatal period who may be at high risk); and 4) were generally representative of other urban and peri-urban primary care health facilities in Mozambique.

### Tools and data collection

Data were collected using the Mini International Neuropsychiatric Interview (MINI 5.0-MZ) adapted to the Mozambican context [24] from the existing Brazilian Portuguese version of the MINI 5.0 [25]. The MINI 5.0-MZ includes a structured psychiatric interview for all common mental disorders and one section for suicide risk screening. The *suicide screening* section consists of dichotomous questions with “no” or “yes” answers, subdivided into two parts, the first referring to the assessment of suicide risk in the past month and the second part assessing suicide attempts over the lifetime. Four questions provided our primary outcome variables: (1) (C2, Suicidal Ideation) In the past month did you think you would be better off dead or wish you were dead? (Portuguese translation: *durante o último mês*, *pensou que seria melhor estar morto ou desejou estar morto?);* (2) (C5, Suicide Plan) In the past month did you make a suicide plan? (Portuguese translation: *durante o último mês, pensou numa maneira de se suicidar?);* (3) (C8, In the past month did you attempt suicide?) (Portuguese translation: *durante o último mês, tentou o suicídio?);* (4) (C9, In your lifetime did you ever make a suicide attempt) (Portuguese translation: *ao longo da sua vida, já fez alguma tentativa de suicídio?).*

Sociodemographic characteristics measured included: age, sex, purpose of visit (outpatient primary care; post-partum consultation; pre-natal consultation), marital status, education, self-reported income earned per month and HIV testing status (HIV positive; HIV negative; never tested). See Table 1 for detailed sub-categories of included sociodemographic variables.

**Table 1:** Demographic and suicidal behavior from the Mozambican adapted MINI International Neuropsychiatric Interview (MINI 5.0—MZ, Suicide Section) administered to 502 Patients in primary care settings in Sofala, Mozambique

Characteristic	Total (N, %)	Suicidal Ideation, Past Month (n, %)	Suicide Plan, Past Month (n, %)	Suicide Attempt, Past Month (n, %)	Suicide Attempt, Lifetime (n, %)
**Total**	**502 (100)**	**63 (12.5)**	**40 (8.0)**	**20 (4.0)**	**25 (5.0)**
**Age, Mean (SD)**	**27.8 (7.4)**	**28.4 (7.9)**	**26.3 (8.5)**	**24.1 (7.5)**	**26.1 (8.0)**
18–24	190 (37.9)	23 (36.5)	20 (50.0)	13 (65.0)	12 (48.0)
25–29	142 (28.3)	15 (23.8)	8 (20.0)	1 (5.0)	3 (12.0)
30–34	63 (12.6)	8 (12.7)	3 (7.5)	2 (10.0)	2 (8.0)
35–39	66 (13.2)	12 (19.1)	7 (17.5)	2 (10.0)	5 (20.0)
40 +	30 (6.0)	4 (6.4)	1 (2.5)	1 (5.0)	1 (4.0)
Don’t know	11 (2.2)	1 (1.6)	1 (2.5)	1 (5.0)	2 (8.0)
**Sex**					
Male	130 (25.9)	15 (23.8)	9 (22.5)	4 (20.0)	5 (20.0)
Female	372 (74.1)	48 (76.2)	31 (77.5)	16 (80.0)	20 (80.0)
**Purpose of visit**					
Outpatient primary care	224 (44.6)	42 (66.7)	29 (72.5)	16 (80.0)	15 (60.0)
Post-partum consultation	138 (27.5)	14 (22.2)	5 (12.5)	2 (10.0)	3 (12.0)
Pre-natal consultation	140 (27.9)	7 (11.1)	6 (15.0)	2 (10.0)	7 (28.0)
**Marital status**					
Married	29 (5.8)	3 (4.8)	2 (5.0)	1 (5.0)	1 (4.0)
Civil union	355 (70.7)	36 (57.1)	22 (55.0)	9 (45.0)	13 (52.0)
Single	100 (20.0)	19 (30.2)	13 (32.5)	8 (40.0)	10 (40.0)
Divorced	4 (0.80)	2 (3.2)	1 (2.5)	0 (0.0)	0 (0.0)
Separated	7 (1.4)	1 (1.6)	1 (2.5)	0 (0.0)	0 (0.0)
Widowed	7 (1.4)	2 (3.2)	1 (2.5)	2 (10.0)	1 (4.0)
**Education**					
No school	2 (0.40)	0 (0.0)	0 (0.0)	0 (0.0)	0 (0.0)
Some primary	74 (14.7)	8 (12.7)	4 (10.0)	2 (10.0)	2 (8.0)
Finished primary	56 (11.2)	6 (9.5)	4 (10.0)	2 (10.0)	4 (16.0)
Some high school	157 (31.3)	24 (38.1)	16 (40.0)	7 (35.0)	3 (12.0)
Finished high school	138 (27.5)	17 (27.0)	13 (32.5)	8 (40.0)	12 (48.0)
Post graduate	65 (13.0)	6 (9.5)	3 (7.5)	1 (5.0)	4 (16.0)
Missing	10 (2.0)	2 (3.2)	0 (0.0)	0 (0.0)	0 (0.0)
**†USD earned per month, Mean (SD)**	**118.5 (147.9)**	**116.2 (123.0)**	**114.5 (108.9)**	**143.5 (143,6)**	**144.9 (124.1)**
Up to 100	259 (58.1)	33 (56.9)	22 (59.5)	10 (55.6)	11 (47.8)
100 to > 250	155 (34.8)	21 (36.2)	12 (32.4)	5 (27.8)	9 (39.1)
250 to > 500	25 (5.6)	3 (5.2)	3 (8.1)	3 (16.7)	3 (13.0)
** = > 500**	7 (1.6)	1 (1.7)	0 (0.0)	0 (0.0)	0 (0.0)
**HIV + **					
No	335 (66.7)	41 (65.1)	27 (67.5)	12 (60.0)	17 (68.0)
Yes	140 (27.9)	20 (31.8)	12 (30.0)	8 (40.0)	8 (32.0)
Never tested	27 (5.4)	2 (3.2)	1 (2.5)	0 (0.0)	0 (0.0)

^†^Converted to USD using the exchange rate of 60 MZN to 1 USD

Patients were recruited from the consultation waiting room of pre-natal, post-partum, and general outpatient consultations from October 2018 to February 2019. While in the waiting room, a trained Health Alliance International research assistant gave a presentation to all individuals sitting in the room and asked them if they would be willing to participate in the study. The research assistant directed interested individuals to a private consultation room to administer the consent form and the short sociodemographic survey in RedCap. Inclusion criteria were 18 years of age or over and agreeing to participate in the study by signing an informed consent form; those who could not read had the consent form read to them. Patients were excluded if they had an acute health condition, a disability impeding their ability to complete the survey, or were less than 18 years of age. Following the sociodemographic survey, a trained Ministry of Health mental health professional (psychologist or psychiatric technician) administered the adapted MINI 5.0-MZ.

### Data analysis procedures

We calculated the prevalence for four key outcomes including: (1) *Suicidal ideation in the past month*; (2) *Suicidal plan in the past month*; (3) *Attempted suicide in the past month*; and (4) *Lifetime attempted suicide*. Regression analyses examined unadjusted (OR) and adjusted odds ratios (aOR) obtained by including all sociodemographic variables simultaneously in one model for each primary outcome. Standard errors were adjusted for clinic clustering using the clustered sandwich estimator. Analyses were conducted using Stata 15 and significance was set at a two-sided alpha value of 0.05.

## Results

### Sociodemographic characteristics

The total number of participants approached was 536, 33 (6.2%) declined to participate, and 1 (0.19%) was excluded due to having age less than 18 years old. As described in Table 1, the majority of the 502 participants were female (74%), with a mean age of 28. Regarding marital status, most of the sample was in a civil union (71%, *n* = 355). Twenty percent were single (20%, *n* = 100). More than half (59%, *n* = 295) of patients had either some high school or had finished high school. The average income per month in US Dollars was $117, which is very high relative to the gross domestic per capita per month of $47 in Mozambique as would be expected given the urban and peri-urban setting, relative to rural settings [26]. Overall, 28% (*n* = 140) of the sample reported testing HIV + , with only 5% (*n* = 27) reporting having never been tested for HIV.

### Prevalence and correlates of suicidal ideation in the past month

The overall prevalence of suicidal ideation in the past month was 12.5% (Table 1). Compared to those attending outpatient primary care visits, those attending post-partum consultations were about 68% less likely to report suicidal ideation in the past month (aOR = 0.32; 95% CI: 0.16—0.65) and those attending pre-natal consultations were about 87% less likely (aOR = 0.13; 95% CI: 0.05—0.35). Women had an elevated but not statistically significant adjusted odds ratio for increased odds of suicidal ideation compared to men (aOR = 2.59; 95% CI: 0.94 – 7.20). Those with a post-graduate education had 50% lower odds of suicidal ideation (aOR = 0.50; 95% CI: 0.39—0.64). Those who had never been tested for HIV had 58% lower odds of reporting suicidal ideation (aOR = 0.42; 95% CI: 0.19—0.89) (Table 2).

**Table 2:** Correlates of Demographic and Suicidal Ideation (Past Month) Suicidal Plan (Past Month) of Mozambican adapted MINI International Neuropsychiatric Interview (MINI 5.0—MZ, Suicide Section) administered to 502 Patients in primary care settings in Sofala, Mozambique

Characteristic	Suicidal Ideation, Past Month, OR (95% CI)	Suicidal Ideation, Past Month, Fully adjusted aOR (95% CI)	Suicide Plan, Past Month, OR (95% CI)	Suicide Plan, Past Month, Fully adjusted aOR (95% CI)
**Age (10 year increase)**	1.12 (0.76, 1.68)	1.05 (0.62, 1.79)	0.72 (0.44, 1.19)	0.61^*^ (0.38, 0.98)
**Sex**				
Male	1 (reference)	1 (reference)	1 (reference)	1 (reference)
Female	1.14 (0.46, 2.80)	2.59 (0.94, 7.20)	1.22 (0.75, 1.99)	2.82^†^ (1.45, 5.51)
**Purpose of visit**				
Outpatient Primary Care	1 (reference)	1 (reference)	1 (reference)	1 (reference)
Post-Partum Consultation	0.49^†^ (0.31, 0.77)	0.32^†^ (0.16, 0.65)	0.25^†^ (0.13, 0.51)	0.09^†^ (0.06, 0.14)
Pre-Natal Consultation	0.23^†^ (0.12, 0.44)	0.13^†^ (0.05, 0.35)	0.30 (0.08, 1.10)	0.13^*^ (0.02, 0.77)
**Marital status**				
Civil Union	1 (reference)	1 (reference)	1 (reference)	1 (reference)
Married	1.02 (0.27, 3.88)	0.98 (0.26, 3.71)	1.12 (0.54, 2.35)	1.46 (0.54, 3.91)
Single	2.08^*^ (1.02, 4.24)	1.39 (0.54, 3.09)	2.26^†^ (1.39, 3.68)	1.19 (0.46, 3.08)
Divorced, Separated, or Widowed	3.41^*^ (1.20, 9.65)	1.06 (0.64, 1.75)	3.03 (0.68, 13.38)	1.18 (0.33, 4.28)
**Education**				
No School or Some primary	0.64 (0.39, 1.05)	0.93 (0.67, 1.30)	0.52^*^ (0.29, 0.96)	0.98 (0.37, 2.60)
Finished Primary	0.65 (0.30, 1.40)	0.68 (0.21, 2.14)	0.73 (0.38, 1.39)	0.72 (0.31, 1.68)
Some High School	1 (reference)	1 (reference)	1 (reference)	1 (reference)
Finished High School	0.76 (0.41, 1.42)	0.64 (0.21, 1.98)	0.98 (0.55, 1.75)	0.71 (0.40, 1.26)
Post graduate	0.55^†^ (0.35, 0.86)	0.50^†^ (0.39, 0.64)	0.46 (0.10, 2.03)	0.39 (0.09, 1.59)
**USD earned per month (50 USD increase)**	0.99 (0.89, 1.11)	1.02 (0.93, 1.11)	0.99 (0.89, 1.10)	1.01 (0.94, 1.10)
**HIV + **				
No	1 (reference)	1 (reference)	1 (reference)	1 (reference)
Yes	1.20 (0.90, 1.58)	0.70 (0.41, 1.19)	1.07 (0.44, 2.57)	0.75 (0.49, 1.16)
Never tested	0.57 (0.29, 1.18)	0.42^*^ (0.19, 0.89)	0.44 (0.02, 8.23)	0.28, 0.01, 5.53)

*N/A* No data for given cell, *Odds* Ratio is undefined

^*^ *p* < .05

^†^ *p* < .01

### Prevalence and correlates of suicidal plan in the past month

The overall prevalence of making a suicide plan in the past month was 8.0% (*n* = 40) (Table 1). Younger individuals had higher odds of a suicide plan in the past month: each 10-year increase in age was associated with 0.61-fold decreased odds in reporting having made a suicide plan (aOR = 0.61; 95% CI: 0.38—0.98). Eighty-seven percent of individuals aged 18–24 who reported suicidal ideation in the past month had made a suicide plan (20/23). Females had 2.8 times the odds of suicide plan in the past month compared to men (aOR = 2.82; 95% CI: 1.45—5.51). Compared to those patients attending outpatient primary care, those attending post-partum consultations or pre-natal consultations had about 91% (aOR = 0.09; 95% CI: 0.06—0.14) and about 87% (aOR = 0.13; 95% CI: 0.02—0.77) lower odds of reporting suicide plan in the past month, respectively (Table 2).

### Prevalence and correlates of attempted suicide in the past month

As described in Table 1, the overall prevalence of a suicide attempt in the past month was 4.0% (*n* = 20). Older individuals had significantly lower odds of a suicide attempt in the past month. Each 10-year increase in age was associated with 91% decreased odds of reporting a suicide attempt in the past month (aOR = 0.09; 95% CI: 0.01–0.69). Fifty-six percent of those aged 18–24 who reported suicidal ideation in the past month had made a suicide attempt in the past month (13/23) and 65% of those with a suicide plan had made a suicide attempt in the past month (13/20). Females had 3.26 times the odds of suicide attempt in the past month (aOR = 3.26; 95% CI: 1.17 – 9.07). Compared to those patients attending outpatient primary care, those attending post-partum consultations or pre-natal consultations had 96% (aOR = 0.04; 95% CI: 0.01—0.27) and 98% (aOR = 0.02; 95% CI: 0.01—0.19) lower odds of suicide plan in the past month, respectively. Individuals reporting to be formally married had 5.5 times the odds of suicide attempt (aOR = 5.5; 95% CI: 3.6–8.5). Each 50 USD per month earned was associated with 12% increased odds of reporting a suicide attempt (aOR = 1.12; 95% CI: 1.08 – 1.15). Individuals testing positive for HIV had 2.2 times the odds of reporting a suicide attempt in the past month (aOR = 2.2; 95% CI: 1.14 – 4.09) (Table 3).

**Table 3:** Correlates of Demographic and Suicide Attempt in the Past Month, Suicide Attempt in the Lifetime of Mozambican adapted MINI International Neuropsychiatric Interview (MINI 5.0-MZ, Suicide Section) administered to 502 Patients in primary care settings in Sofala, Mozambique

Characteristic	Suicide Attempt, Past Month, OR (95% CI)	Suicide Attempt, Past Month, adjusted aOR (95% CI)	Suicide Attempt, Lifetime, OR (95% CI)	Suicide Attempt, Lifetime, adjusted aOR (95% CI)
**Age (10 year increase)**	0.36^†^ (0.17, 0.79)	0.09^*^ (0.01, 0.69)	0.69 (0.31, 1.57)	0.70 (0.40, 1.22)
**Sex**				
Male	1 (reference)	1 (reference)	1 (reference)	1 (reference)
Female	1.42 (0.40, 5.05)	3.26^†^ (1.17, 9.07)	1.42 (0.49, 4.1)	2.94 (0.88, 9.8)
**Purpose of visit**				
Outpatient primary care	1 (reference)	1 (reference)	1 (reference)	1 (reference)
Post-partum consultation	0.19^†^ (0.12, 0.31)	0.04^†^ (0.01, 0.27)	0.31^†^ (0.18, 0.53)	0.10^*^ (0.01, 0.92)
Pre-natal consultation	0.19^*^ (0.05, 0.71)	0.02^†^ (0.01, 0.19)	0.73 (0.25, 2.2)	0.59 (0.15, 2.35)
**Marital status**				
Civil union	1 (reference)	1 (reference)	1 (reference)	1 (reference)
Married	1.37 (0.59, 3.2)	5.5^†^ (3.6, 8.5)	0.94 (0.17, 5.18)	0.55 (0.06, 4.96)
Single	3.34^†^ (1.79, 6.25)	0.57 (0.24, 1.37)	2.92^†^ (1.51, 5.65)	1.73 (0.61, 4.95)
Divorced, Separated, or Widowed	4.81 (0.25, 90.98)	1.0 (0.03, 33.2)	1.55 (0.12, 19.40)	N/A
**Education**				
No School or Some primary	0.62 (0.28, 1.35)	2.30 (0.56, 9.36)	1.48 (0.18, 12.0)	0.99 (0.08, 12.34)
Finished primary	0.85 (0.40, 1.80)	0.34^†^ (0.28, 0.41)	4.20^†^ (2.01, 8.80)	3.77^*^ (1.34, 10.58)
Some high school	1 (reference)	1 (reference)	1 (reference)	1 (reference)
Finished high school	1.41^†^ (1.07, 1.84)	0.86 (0.55, 1.35)	5.21^†^ (2.48, 10.92)	2.74^*^ (1.06, 7.12)
Post graduate	0.36 (0.09, 1.45)	0.20 (0.03, 1.22)	3.58 (2.8, 4.6)	2.86^†^ (2.26, 3.62)
**USD earned per month (50 USD change)**	1.04 (0.99, 1.09)	1.12^†^ (1.08, 1.15)	1.05† (1.02, 1.08)	1.06 (0.99, 1.13)
**HIV + **				
No	1 (reference)	1 (reference)	1 (reference)	1 (reference)
Yes	1.63 (0.35, 7.56)	2.16^*^ (1.14, 4.09)	1.13 (0.41, 3.10)	1.29 (0.78, 2.14)
Never tested	N/A	N/A	N/A	N/A

*N/A* No data for given cell, *Odds* Ratio is undefined

^*^ *p* < .05

^†^ *p* < .01

### Prevalence and correlates of lifetime attempted suicide

As described in Table 1, the overall prevalence of lifetime suicide attempts was 5.0% (*n* = 25). Compared to those patients attending outpatient primary care, those attending post-partum consultations had 90% lower odds of lifetime suicide attempt (aOR = 0.10; 95% CI: 0.01 – 0.92). Females had an elevated point estimate but not statistically significant increased odds of lifetime suicide attempt (aOR = 2.9; 95% CI: 0.88 – 9.8). Compared to those who had some high school, those who finished primary, finished high school, or had post graduate education had elevated odds of lifetime suicide attempt (Table 3).

## Discussion

Suicidal behavior appears to be common among primary care attendees in Mozambique. Overall, one in every eight (12.5%) patients expressed suicidal ideation in the past month. This increased to one in five (18.8%) among patients attending outpatient primary care. Sixty three percent of those with suicidal ideation in the past month had made a suicide plan (40/63) and 50% of those who had a suicide plan had made a suicide attempt in the past month (20/40). These numbers were particularly elevated when considering younger individuals aged 18–24. Over 85% of individuals aged 18–24 who had suicidal ideation made a suicide plan (20/23) and 65% who made a suicide plan made a suicide attempt (13/20). Together, these data show the opportunity for intervening in the prevention of suicide attempts, especially for younger individuals attending primary care visits in Mozambique. While lower than outpatient primary care, those attending post-partum consultations and pre-natal consultations still had high rates of suicidal ideation at 10% (14/138) and 5% (7/140) of attendees, respectively. In terms of high-risk groups for suicidal behavior in primary care, our study identified younger individuals, females, individuals attending outpatient primary care, and people living with HIV/AIDS at increased risk of suicidal behavior. These high rates of suicidal behavior among primary care patients – including suicide attempts – highlights the urgent need to integrate screening and effective prevention approaches for suicidal behavior into primary care.

The rates of suicidal ideation in the past month among primary care attendees in the present study (12.5%; 63/502) are similar [13] to a recent study in Kenya finding past month suicidal ideation of 20%, suicide plan of 10%, and suicide attempt of 4% [15]. Comparing our findings to previous community-level samples, the present study suggests that suicidal ideation may be over twice as prevalent among primary care attendees compared to the general population in community settings in Mozambique [19] as observed in high income countries (HICs) [27]. The risk factors of female gender, younger age, and being positive for HIV for suicidal behavior are in line with previous cross-national studies in LMICs [13, 28, 29]. Another study done in China found that PLWHA are at increased risk for suicidal ideation, especially when those patients present with depression, low self-esteem, and poor social support [30]. In another study done in an African country, PLWHA who were unmarried, unemployed, with HIV status non-disclosure were more at risk for presenting suicidal ideation [31].

The dramatically increased risk for suicidal ideation to progress to suicide plan and suicide attempt among younger individuals in our setting bears additional mention. While individuals aged 18–24 made up only 37.8% of our sample (190/502), they accounted for 50% of suicide plans made (20/40), and 65% of suicide attempts (13/20). The fact that 56% of individuals aged 18–24 with suicidal ideation in the past month also attempted suicide in the past month shows the potential for early and frequent screening for suicidal ideation in primary care as a potential avenue for preventing suicide attempts and deaths. In addition, individuals living with HIV were not over-represented in terms of suicidal ideation compared to our overall sample – however – of those individuals living with HIV who did express suicidal ideation in the past month, 60% had made a suicide plan and 67% of those with a suicide plan had attempted suicide in the past month (Table 1). Thus, the integration of screening for suicidal ideation for PLWHA in Mozambique and the recognition that without linkage to effective care over 40% of these individuals may go on to attempt suicide within the next month should be considered an urgent priority. This result is in line with the findings found in a Chinese study where suicidal ideation was very common in PLWHA and emphasizing the need for early screening for this problem in this high-risk group and urgent provision of adequate psychosocial support [30].

The present study is not without its limitations. First, this study was conducted in three primary care health centers in the central region in Mozambique and thus may not generalize to other regions in Mozambique. Second, our study sampling method recruited willing participants from primary care waiting rooms and thus was a sample of convenience. Notwithstanding these limitations, our study has a number of notable strengths. To our knowledge this is the first evaluation of suicidal behavior among primary care attendees in Mozambique. Patients were sourced from routine primary care centers run by the Ministry of Health which constitute usual care for general clinical care in the country. We utilized a structured clinical questionnaire adapted to the local context and delivered by local Mozambican psychologists and psychiatric technicians working at the primary care centers of interest, thus increasing the quality of the data.

## Conclusion

In this study suicidal behavior was common among individuals seeking primary healthcare in Mozambique. Females, younger individuals, and people living with HIV may be at particularly elevated risk for suicidal behavior in this context. Younger individuals made up only 38% of the sample yet represented 65% of suicide attempts. Our finding that over 56% of individuals aged 18–24 reporting suicidal ideation in the past month had a suicide attempt shows the urgent need to provide routine screening for suicidal ideation during primary care contacts and link individuals to effective prevention interventions. The Ministry of Health in Mozambique should focus on strategies for integrating suicide screening and prevention activities in primary healthcare and could focus specific efforts on younger individuals, women, and those living with chronic illnesses such as HIV/AIDS.

## Data Availability

The datasets used and/or analyzed during the current study are available from Health Alliance International, Mozambique under reasonable request addressed to corresponding author Vasco FJ Cumbe (vcumbe@gmail.com) or to Bradley H. Wagenaar (wagenaarb@gmail.com).

## Abbreviations

AIDS: Acquired Immunodeficiency Syndrome
aOR: Adjusted Odds Ratio
CI: Confidence Interval
HIV: Human Immunodeficiency Virus
INE: Instituto Nacional de Estatística (National Institute of Statistician)
LMICs: Low-and Middle-Income Countries
MINI: Mini International Neuropsychiatric Interview
OR: Odds Ratio
HIC: High Income Countries
PHC: Primary Health Care
PLWHA: People Living With HIV
SA: Suicide Attempt
SDGs: Sustainable Development Goals
SI: Suicidal Ideation
SP: Suicidal Plan
SD: Standard Deviation
MZ: Mozambique
N/A: Not Applicable
WHO: World Health Organization

## References

[1] Global Health Estimates 2019: Deaths by Cause, Age, Sex, by Country, 2000–2019 and by Region. Geneva, World Health Organization, 2020. Geneva; 2020.

[2] Roth GA, Abate D, Abate KH, Abay SM, Abbafati C, Abbasi N, et al. Global, regional, and national age-sex-specific mortality for 282 causes of death in 195 countries and territories, 1980–2017: a systematic analysis for the Global Burden of Disease Study 2017. Lancet. 2018;392(10159):1736–88. 10.1016/S0140-6736(18)32203-7 PMC6227606 30496103

[3] Suicide in the World: Global Health Estimates. World Health Organization; 2019. 2019. Available from: http://apps.who.int/bookorders.

[4] United Nations. The Sustainable Development Goals Report. 2017. 2018. [cited 2022 Apr 21]. Available from: https://unstats.un.org/sdgs/files/report/2017/thesustainabledevelopmentgoalsreport2017.pdf.

[5] World Health Organization. Mental Health Action Plan 2013–2030. 2013 [cited 2021 Jun 14]. Available from: http://www.emro.who.int/mnh/mental-health-action-plan/index.html.

[6] Naghavi M (2019). Global, regional, and national burden of suicide mortality 1990 to 2016: systematic analysis for the Global Burden of Disease Study 2016. BMJ.

[7] Metrics GH. Global, regional, and national incidence, prevalence, and years lived with disability for 354 diseases and injuries for 195 countries and territories, 1990–2017: a systematic analysis for the Global Burden of Disease Study 2017 (The Lancet (2018) 392(1015)). Lancet. 2019;393(10190): e44. 10.1016/S0140-6736(18)32279-7 PMC6227754 30496104

[8] World Health Organization (2014). Preventing Suicide: A Global Imperative.

[9] Sher L. The impact of the COVID-19 pandemic on suicide rates. Vol. 113, QJM. Oxford University Press; 2020. p. 707–12. 10.1093/qjmed/hcaa202 PMC7313777 32539153

[10] Bertolote JM, Fleischmann A. A global perspective in the epidemiology of suicide. Suicidologi. 2015;7(2). [cited 2021 May 9]. Available from: https://www.journals.uio.no/index.php/suicidologi/article/view/2330.

[11] Mishara BL, Weisstub DN. The legal status of suicide: A global review. Int J Law Psychiatry. 2016;44:54–74. 10.1016/j.ijlp.2015.08.032 26375452

[12] World Health Organization. WHO Suicide. Fact sheet. Geneva; 2019. Available from: https://www.who.int/news-room/fact-sheets/detail/suicide.

[13] Jordans M, Rathod S, Fekadu A, Medhin G, Kigozi F, Kohrt B, et al. Suicidal ideation and behaviour among community and health care seeking populations in five low- and middle-income countries: A cross-sectional study. Epidemiol Psychiatr Sci. 2018;27(4):393–402. 10.1017/S2045796017000038 PMC5559346 28202089

[14] Oneib B, Sabir M, Otheman Y, Abda N, Ouanass A. Suicidal ideations, plans and attempts in primary care in Morocco: cross-sectional study of consultants at primary health care system in Morocco. Pan Afr Med J. 2016;24. Available from: http://www.panafrican-med-journal.com/content/article/24/274/full/. 10.11604/pamj.2016.24.274.9060 PMC5267922 28154629

[15] Ongeri L, McCulloch CE, Neylan TC, Bukusi E, Macfarlane SB, Othieno C (2018). Suicidality and associated risk factors in outpatients attending a general medical facility in rural Kenya. J Affect Disord.

[16] Sherr K, Fernandes Q, Kanté AM, Bawah A, Condo J, Mutale W, et al. Measuring health systems strength and its impact: Experiences from the African Health Initiative. BMC Health Serv Res. 2017;17(Suppl 3):827. 10.1186/s12913-017-2658-5 PMC5763472 29297341

[17] Unicef Mozambique. Ciclone Idai e Kenneth _ UNICEF Mozambique. 2019. [cited 2021 May 10]. Available from: https://www.unicef.org/mozambique/ciclone-idai-e-kenneth.

[18] Wagenaar BH, Raunig-Berhó M, Cumbe V, Rao D, Napúa M, Sherr K (2016). Suicide Attempts and Deaths in Sofala, Mozambique, From 2011 to 2014. Crisis.

[19] Halsted S, Ásbjörnsdóttir KH, Wagenaar BH, Cumbe V, Augusto O, Gimbel S, et al. Depressive symptoms, suicidal ideation, and mental health care-seeking in central Mozambique. Soc Psychiatry Psychiatr Epidemiol. 2019;54(12):1519–33. 10.1007/s00127-019-01746-2 PMC7050264 31317245

[20] Vázquez-Machado A, Mukamutara J, Jimmy Hirzel P, Granma B. Epidemiología del intento suicida en el Hospital Central de Nampula, Mozambique. Epidemiology of suicide attempts at Nampula Central Hospital, Mozambique. Rev Neuropsiquiatr. 201982:117–24.

[21] Instituto Nacional de Estatística. Delegação Provincial de Sofala. Anuário Provincial de Estatística de Sofala 2019. 2019. Available from: www.ine.gov.mz.

[22] Direcção Provincial de Saúde de Sofala. Departamento de Saúde Pública. Relatório Anual de Saúde Mental, 2020. Moçambique, Beira. 2021.

[23] Fernandes QF, Wagennar BH, Anselmi L, Pfeiffer J, Gloyd S, Sherr K (2008). Effects of Health-System Strengthening on Under-5 Infant, and Neonatal Mortality: 11-Year provincial-Level Time-Series Analyses in Mozambique. Bone.

[24] Cumbe VFJ, Muanido A, Manaca MN, Fumo H, Chiruca P, Hicks L (2020). Validity and item response theory properties of the Patient Health Questionnaire-9 for primary care depression screening in Mozambique (PHQ-9-MZ). BMC Psychiatry.

[25] Amorim P (2000). Mini International Neuropsychiatric Interview (MINI): validação de entrevista breve para diagnóstico de transtornos mentais. Rev Bras Psiquiatr.

[26] Mozambique GDP Per Capita 1980–2020. Mozambique GDP Per Capita: 1980–2020 Data/2021–2023 Forecast/Historical/Chart. 2021. [cited 2021 Aug 13]. Available from: https://tradingeconomics.com/mozambique/gdp-per-capita.

[27] Biswas T, Scott JG, Munir K, Renzaho AMN, Rawal LB, Baxter J, et al. Global variation in the prevalence of suicidal ideation, anxiety, and their correlates among adolescents: A population based study of 82 countries. EClinicalMedicine. 2020;24:100395. 10.1016/j.eclinm.2020.100395 PMC7525128 33015595

[28] Vijayakumar L, Nagaraj K, Pirkis J, Whiteford H (2005). Suicide in Developing Countries (1). Crisis.

[29] Nock MK, Borges G, Bromet EJ, Alonso J, Angermeyer M, Beautrais A (2008). Cross-National Prevalence and Risk Factors for Suicidal Ideation, Plans, and Attempts. Br J Psychiatry.

[30] Wang W, Xiao C, Yao X, Yang Y, Yan H, Li S. Psychosocial health and suicidal ideation among people living with HIV/AIDS: A cross-sectional study in Nanjing, China. PLoS One. 2018;13(2):e0192940. 10.1371/journal.pone.0192940 PMC5823403 29470532

[31] Ogundipe OA, Olagunju AT, Adeyemi JD (2015). Suicidal Ideation among Attendees of a West African HIV Clinic. Arch Suicide Res.

